# High-pressure protein crystal structure analysis of *Escherichia coli* dihydrofolate reductase complexed with folate and NADP^+^

**DOI:** 10.1107/S2059798318009397

**Published:** 2018-09-03

**Authors:** Takayuki Nagae, Hiroyuki Yamada, Nobuhisa Watanabe

**Affiliations:** a Synchrotron Radiation Research Center, Nagoya University, Chikusa, Nagoya 464-8603, Japan; b Venture Business Laboratory, Nagoya University, Chikusa, Nagoya 464-8603, Japan; c Graduate School of Engineering, Nagoya University, Chikusa, Nagoya 464-8603, Japan

**Keywords:** high pressure, diamond anvil cell, dihydrofolate reductase

## Abstract

A high-pressure crystallographic study was conducted on *Escherichia coli* dihydrofolate reductase (ecDHFR) complexed with folate and NADP^+^ in crystal forms containing both the open and closed conformations of the M20 loop under high-pressure conditions of up to 800 MPa. At pressures between 270 and 500 MPa the crystal form containing the open conformation exhibited a phase transition from *P*2_1_ to *C*2. Several structural changes in ecDHFR were observed at high pressure that were also accompanied by structural changes in the NADP^+^ cofactor and the hydration structure. In the crystal form with the closed conformation the M20 loop moved as the pressure changed, with accompanying conformational changes around the active site, including NADP^+^ and folate. These movements were consistent with the suggested hypothesis that movement of the M20 loop was necessary for ecDHFR to catalyze the reaction. In the crystal form with the open conformation the nicotinamide ring of the NADP^+^ cofactor undergoes a large flip as an intermediate step in the reaction, despite being in a crystalline state. Furthermore, observation of the water molecules between Arg57 and folate elucidated an early step in the substrate-binding pathway. These results demonstrate the possibility of using high-pressure protein crystallography as a method to capture high-energy substates or transient structures related to the protein reaction cycle.

## Abstract 2

High-pressure crystal structure analysis of *E. coli* dihydrofolate reductase (ecDHFR) complexed with folate and NADP^+^ was performed up to 800 MPa. A pressure-induced phase transition of monoclinic ecDHFR crystals and structural changes closely related to its reaction mechanism were observed.

## 1. Introduction   

Dynamic structural information is important in understanding the true nature of proteins in their native biological systems. Enzymes, for example, alter their conformations along their reaction pathways, and time-resolved crystallography is a prospective approach to studying the reaction mechanisms of proteins. It is now possible to achieve even subpicosecond time resolution when using X-ray free-electron lasers (XFELs; Schlichting, 2015 ▸). XFELs are a very enticing field of study, but access to such experimental equipment is not easy for many crystallographers worldwide. In the crystallographic study of proteins to date, the atomic temperature factors in protein molecular domains have been used to express protein dynamics as a positional uncertainty or thermal displacement. Recently, multi-conformer models have been utilized to describe protein-molecule fluctuations when high-resolution crystal structures, particularly at room temperature, can be obtained (Fenwick *et al.*, 2014 ▸; Keedy *et al.*, 2014 ▸). However, it is only possible to obtain information about the conformational motions of proteins near their ground state using such approaches, and it is difficult to study conformational changes of proteins at different stages along their reaction pathways.

Here, we pursue an alternate route to studying protein dynamics using a high-pressure environment. In accordance with Le Chatelier’s principle, high pressure initiates structural changes in proteins by shifting the equilibrium to reduce the partial molar volume of the system, thereby enabling the observation of metastable structures or high-energy conformational substates (Akasaka, 2006 ▸). Such substates are difficult to study because of their low abundance under physiological conditions, but they frequently provide valuable information about the enzymatic reaction mechanism (Collins *et al.*, 2011 ▸; Fourme *et al.*, 2012 ▸). X-ray crystallography is superior to the other spectroscopic methods because it can be used to obtain straightforward three-dimensional structures of proteins and the surrounding water structure. High-pressure protein crystallography (HPPX) with a diamond anvil cell (DAC) was developed using synchrotron radiation (Fourme *et al.*, 2001 ▸) and has been used to study the pressure-response behaviors of proteins, including the compressibility of protein molecules and hydration-structure changes, such as water penetration into hydrophobic cavities (Collins *et al.*, 2005 ▸; Ascone *et al.*, 2010 ▸; Nagae *et al.*, 2012 ▸). At present, our previous study on hen egg-white lysozyme appears to be the only example to discuss the high-energy conformational substates of catalytic residues in an enzyme observed using HPPX (Yamada *et al.*, 2015 ▸).

Dihydrofolate reductase (DHFR) is a key enzyme that is essential for the synthesis of purines, thymidylic acids and some amino acids. DHFR catalyzes the reduction of 7,8-dihydrofolate to 5,6,7,8-tetrahydrofolate using NADPH as a cofactor and plays an influential role in folate (FOL) management in many species. The complete kinetic scheme for DHFR from *Escherichia coli* (ecDHFR) is available (Fierke *et al.*, 1987 ▸), and the reaction mechanism of ecDHFR has been studied in detail with cofactor and substrate analogs using X-ray crystallography (Sawaya & Kraut, 1997 ▸). The structural dynamics of ecDHFR have also been investigated using nuclear magnetic resonance (NMR), computational simulations and tryptophan fluorescence probing. In total, more than 90 structures have been deposited in the Protein Data Bank (PDB; Falzone *et al.*, 1994 ▸; Kitahara *et al.*, 2000 ▸; Schnell *et al.*, 2004 ▸; McElheny *et al.*, 2005 ▸; Boehr *et al.*, 2006 ▸, 2010 ▸; Hanoian *et al.*, 2015 ▸; Kohen, 2015 ▸; Reddish *et al.*, 2016 ▸; Abdizadeh *et al.*, 2017 ▸; Huang *et al.*, 2017 ▸; Oyen *et al.*, 2017 ▸).

These studies indicate that ecDHFR may be a good benchmark test system for evaluating the potential of HPPX. Previous structural and kinetic studies have suggested that this enzyme contains three functional loops, termed the M20 loop, FG loop and GH loop. The M20 loop of ecDHFR can adopt three conformations (open, closed and occluded). In the open conformation the M20 loop plays a role in the binding and release of ligands. In the closed conformation the M20 loop interacts with the cofactor and contributes to the chemical reaction step. In the occluded conformation the M20 loop prevents the access of the cofactor to the active site; the M20 loop undergoes large-scale motion between the three conformations depending on the stage in the enzymatic cycle (Sawaya & Kraut, 1997 ▸; Miller *et al.*, 2001 ▸; Osborne *et al.*, 2001 ▸; Schnell *et al.*, 2004 ▸). The coupled motions of the functional loops have been studied by a variety of techniques, including molecular-dynamics and quantum-mechanical/molecular-mechanical (QM/MM) simulations (Radkiewicz & Brooks, 2000 ▸; Agarwal *et al.*, 2002 ▸; Hammes-Schiffer & Benkovic, 2006 ▸; Arora & Brooks, 2009 ▸). Motions of the nicotinamide ring of the NAPDH cofactor into and out of the active site are also coupled to the conformation of the M20 loop (McElheny *et al.*, 2005 ▸).

Here, we report the HPPX study of ecDHFR using a DAC. We determined the structure of ecDHFR in complex with FOL and NADP^+^ under pressures varying between 0.1 and 800 MPa. The ecDHFR–FOL–NADP^+^ ternary complex is a pseudo-Michaelis complex that is valuable for investigation of the catalytic mechanism of DHFR (Sawaya & Kraut, 1997 ▸). The ecDHFR crystal in this study contains both the open and closed conformations of the M20 loop. By comparing the structures at ambient-to-high pressures, conformational changes were observed for ecDHFR, its cofactor and FOL, and the surrounding water molecules.

## 2. Materials and methods   

### 2.1. Overexpression and purification of ecDHFR   

EcDHFR was overexpressed in *E. coli* BL21(DE3) cells (Agilent Technologies) after transformation with the pfolA-ec vector. This vector carried an *E. coli folA* gene with an N-terminal His tag and thrombin site, which was inserted into the NdeI–XhoI sites of pET-28b (Novagen). The bacterial culture was grown to an OD_600_ of 0.6 at 310 K in LB medium containing 50 mg l^−1^ kanamycin. Expression of ecDHFR was induced with 0.5 m*M* isopropyl β-d-1-thiogalactopyranoside. The cells were cultivated for 4 h after induction and harvested by centrifugation. To remove residual broth, the cell pellet was washed with 25 m*M* Tris–HCl buffer pH 7.5 containing 150 m*M* NaCl and stored frozen at 193 K. The cells were resuspended in 25 m*M* Tris–HCl buffer pH 7.5 containing 150 m*M* NaCl and 0.1 mg ml^−1^ lysozyme and were then disrupted by sonication. The lysate was cleared by centrifugation at 20 000*g* for 30 min and the supernatant was loaded onto a nickel–nitrilotriacetic acid (Ni–NTA)–agarose (Qiagen) column equilibrated with 25 m*M* Tris–HCl buffer pH 7.5 containing 150 m*M* NaCl and 5 m*M* β-mercaptoethanol (β-ME). The column was washed with 25 m*M* Tris–HCl buffer pH 7.5 containing 300 m*M* NaCl, 20 m*M* imidazole and 5 m*M* β-ME. The bound proteins were eluted with 25 m*M* Tris–HCl buffer pH 7.5 containing 300 m*M* NaCl, 300 m*M* imidazole and 5 m*M* β-ME. The sample was dialyzed against 25 m*M* Tris–HCl buffer pH 7.5 containing 150 m*M* NaCl and 5 m*M* β-ME to remove imidazole. The His tag was removed using thrombin (GE Healthcare) by incubation for 20 h at 295 K. ecDHFR was separated from the His tag by a second passage over the same column. ecDHFR was further purified by gel filtration on a HiLoad Superdex 75 26/600 prep-grade column (GE Healthcare) equilibrated with 25 m*M* Tris–HCl buffer pH 7.5 containing 150 m*M* NaCl and 5 m*M* β-ME. The collected protein fractions were dialyzed against 10 m*M* Tris–HCl buffer pH 7.5 and concentrated to 60 mg ml^−1^.

### 2.2. Crystallization of ecDHFR   

We used two slightly modified crystallization conditions to produce two types of ecDHFR crystals based on the work of Sawaya & Kraut (1997 ▸). Crystals of the ecDHFR–FOL–NADP^+^ complex with a closed M20 loop conformation (M20-closed crystals) were obtained at 277 K *via* hanging-drop vapor diffusion with a tripartite mixture (40 mg ml^−1^ ecDHFR, 5 m*M* FOL and 5 m*M* NADP^+^ in 10 m*M* Tris–HCl pH 7.5) and a reservoir solution consisting of 18%(*w*/*v*) polyethylene glycol (PEG) 400, 100 m*M* calcium chloride, 20 m*M* imidazole buffer pH 6.0. Crystals with a M20 loop-open conformation (M20-open crystals) were obtained using the same tripartite mixture and a reservoir solution consisting of 32%(*w*/*v*) PEG 6000, 10 m*M* calcium chloride, 100 m*M* imidazole buffer pH 6.5. Large single crystals for high-pressure studies were obtained by the microseeding technique as described previously (Wan, Kovalevsky *et al.*, 2014 ▸). The M20-closed crystal, space group *P*2_1_2_1_2_1_, grew to typical dimensions of 0.2 × 0.5 × 0.3 mm within a week. The M20-open crystal, space group *P*2_1_, grew to typical dimensions of 0.2 × 0.5 × 0.05 mm within 3–4 d.

### 2.3. Data collection   

As a preparatory step for data collection, the crystals were transferred into a pressure-medium solution in which the crystals would not degrade or dissolve at high pressure. The M20-closed crystals were soaked in 40%(*w*/*v*) PEG 400, 100 m*M* calcium chloride, 20 m*M* imidazole buffer pH 6.0 overnight and then transferred into a solution of 50%(*w*/*v*) PEG 400 immediately prior to conducting the experiment. The M20-open crystals were soaked in 35%(*w*/*v*) PEG 6000, 10 m*M* calcium chloride, 100 m*M* imidazole buffer pH 6.5. HPPX experiments using a DAC were performed on Nagoya University beamline BL2S1 at the Aichi Synchrotron Radiation Center (AichiSR; Watanabe *et al.*, 2017 ▸) and beamline NW12A at the Photon Factory (PF-AR; Chavas *et al.*, 2013 ▸), Japan. The X-ray wavelengths were 0.71 or 0.75 Å, the shortest practical wavelengths of the beamlines, to reduce the absorption by the two diamond anvils of the DAC and to cover the maximum resolution restricted by the open angle of the DAC. The crystals were mounted in a DAC sample chamber and gradually compressed to the desired pressure. To stabilize the crystal positions in the chamber, the crystals were placed with tied cigarette-filter fibers (Nagae *et al.*, 2012 ▸). Several crystals were used for data collection at each pressure point to produce a complete data set without severe radiation damage, since all measurements were conducted at room temperature. The pressure in the sample chamber was verified before and after X-ray experiments using the wavelength shift of ruby fluorescence (Zha *et al.*, 2000 ▸). Diffraction data sets were collected from the M20-closed crystals at pressures of up to 800 MPa. The M20-open crystals diffracted reasonably well at pressures as high as 750 MPa, but diffraction spots could not be detected at 800 MPa.

Data collection from the M20-open crystals at atmospheric pressure was performed with a crystal mounted in a glass capillary using a Rigaku FR-E Cu *K*α X-ray source equipped with a Rigaku R-AXIS VII detector. The data-collection conditions are summarized in Table 1 ▸.

### 2.4. Data processing and structure analysis   

The diffraction patterns were indexed, integrated and scaled using *XDS* (Kabsch, 2010 ▸) or *HKL*-2000 (Otwinowski & Minor, 1997 ▸). During integration, the mosaicity was used to detect radiation damage to the crystals. Only frames without serious damage were used for structural analysis. The structures of the M20-closed and M20-open crystals at atmospheric pressure were solved by *MOLREP* (Vagin & Teplyakov, 2010 ▸) as implemented in *CCP*4 (Winn *et al.*, 2011 ▸) with the structures of PDB entries 1rx2 and 1rb2 (Sawaya & Kraut, 1997 ▸) as search models, respectively. Structures at high pressure were refined using the structures at atmospheric pressure as starting models. *REFMAC* (Murshudov *et al.*, 2011 ▸) or *PHENIX* (Adams *et al.*, 2010 ▸) was used for structure refinement, and manual correction of the structures was performed using *Coot* (Emsley & Cowtan, 2004 ▸). *MOLREP* was also used when a crystallographic phase transition occurred for the M20-open crystal. Water molecules were added with *ARP*/*wARP* (Lamzin & Wilson, 1993 ▸) or *PHENIX*. Parameters for data processing and structure refinement are also listed in Table 1 ▸. Atomic coordinates and structure factors over a series of pressures have been deposited in the PDB as entries 5z6f, 5z6j, 5z6k, 5z6l and 5z6m for the M20-closed crystal at 0.1, 220, 400, 650 and 800 MPa, respectively, and 4x5f, 4x5g, 4x5h, 4x5i and 4x5j for the M20-open crystal at 0.1, 270, 500, 660 and 750 MPa, respectively.

Calculations of solvent-excluded volumes in ecDHFR–FOL–NADP^+^ were performed using *VOIDOO* with a probe radius of 1.4 Å (Kleywegt & Jones, 1994 ▸). The volumes of the internal cavities were calculated using *CASTp* with a probe radius of 1.2 Å (Dundas *et al.*, 2006 ▸). Figures were drawn using *PyMOL* v.1.8 (http://www.pymol.org).

## 3. Results   

We determined the high-pressure crystal structure of the ecDHFR–FOL–NADP^+^ ternary complex at 1.7–2.2 Å resolution with the M20 loop adopting both closed and open conformations. The crystals diffracted sufficiently to solve the structures at pressures as high as 800 and 750 MPa, respectively. However, diffraction disappeared at higher pressures. In the M20-open crystal, a crystal-to-crystal phase transition occurred between 270 and 500 MPa. The space group of the crystal changed from *P*2_1_ to *C*2. The relatively high mosaicity of the crystal above 500 MPa indicated the effect of the transition process (Table 1 ▸). Hence, for the open conformation two independent molecules, *A* and *B*, existed in the asymmetric unit of the *P*2_1_ crystal at lower pressures. Because little difference existed between the two molecules, only the *B* chain is discussed and presented in the figures.

### 3.1. Internal cavities   

**Figure 1:**
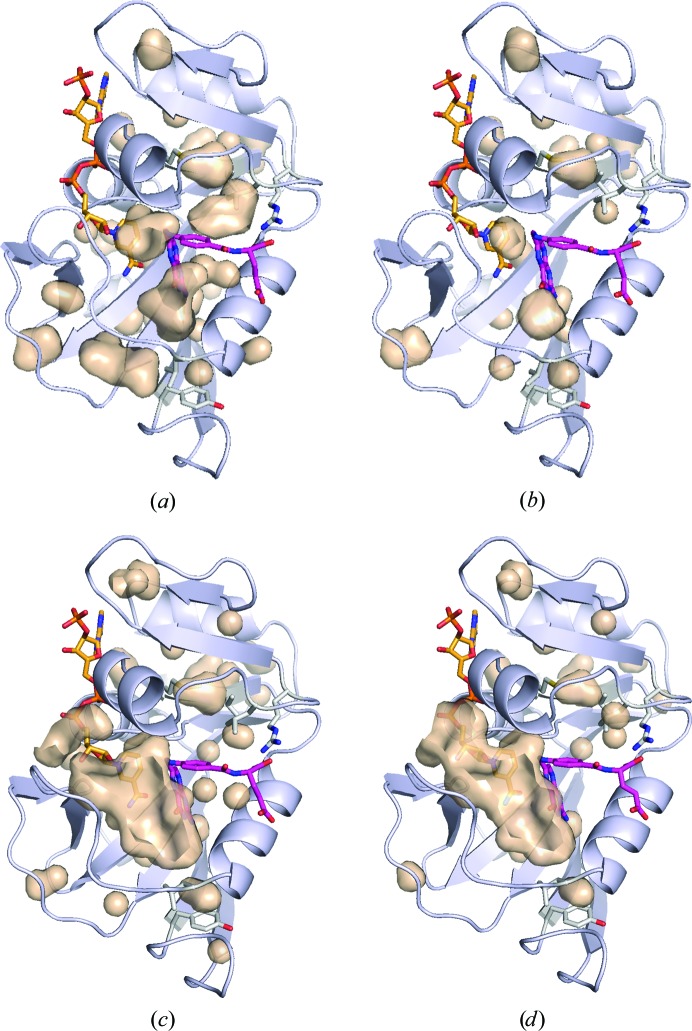
Internal cavities of ecDHFR. (*a*) 0.1 and (*b*) 220 MPa structures of the M20-closed crystal and (*c*) 0.1 and (*d*) 270 MPa structures of the M20-open crystal. Cavities were drawn with the surface-cavity mode of *PyMOL* using a solvent radius of 1.2 Å. NADP^+^ and FOL are treated as components of the protein molecule, and water molecules are ignored. NADP^+^, FOL and several residues referred to in Fig. 3 ▸ are shown as stick models. For the M20-open crystal, (*c*) and (*d*) correspond to the *B* chain in the asymmetric unit.

**Figure 2:**
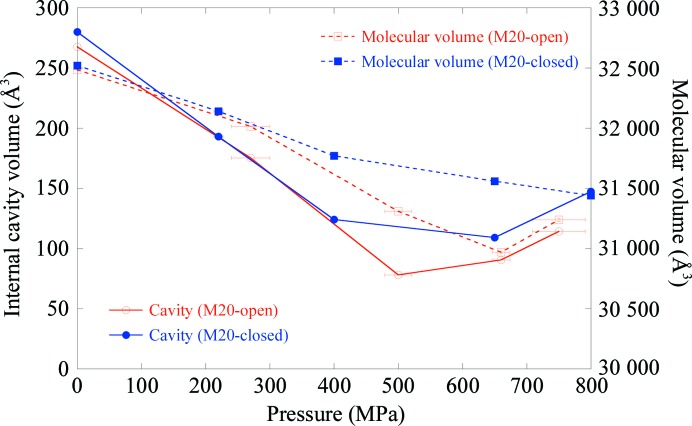
Variations of the total volume of internal cavities and the solvent-excluded volumes of M20-closed and M20-open ecDHFR crystals as a function of pressure. The volumes for the M20-open crystal at 0.1 and 270 MPa are the average for the two molecules in the asymmetric unit of the *P*2_1_ cell. The errors in the volumes are smaller than the symbol sizes.

It is well known that several hydrophobic internal cavities exist in ecDHFR (Gekko, 2002 ▸). In this study, we observed that most of the cavities were compressed as the pressure increased. Examples of cavity changes with pressure are shown in Fig. 1 ▸. The total volume of the ecDHFR internal cavities decreased with pressure (Fig. 2 ▸). In response to this, the solvent-excluded volume of ecDHFR also decreased with pressure. The volume of the M20-closed crystal was 32 520 Å^3^ at atmospheric pressure and was reduced to 31 440 Å^3^ at 800 MPa. That of the M20-open crystal decreased from 32 485 Å^3^ at atmospheric pressure to 30 970 Å^3^ at 660 MPa. The volume increased slightly to 31 240 Å^3^ at 750 MPa, where the volume was averaged for the two molecules in the asymmetric *P*2_1_ unit cell. The average compressibility calculated using the volume of the molecule was 4.6 × 10^−2^ GPa^−1^ for the M20-closed crystal and 6.9 × 10^−2^ GPa^−1^ for the M20-open crystal, which were comparable with those of other proteins previously reported using HPPX (Kundrot & Richards, 1987 ▸; Ascone *et al.*, 2010 ▸; Nagae *et al.*, 2012 ▸; Yamada *et al.*, 2015 ▸).

**Figure 3:**
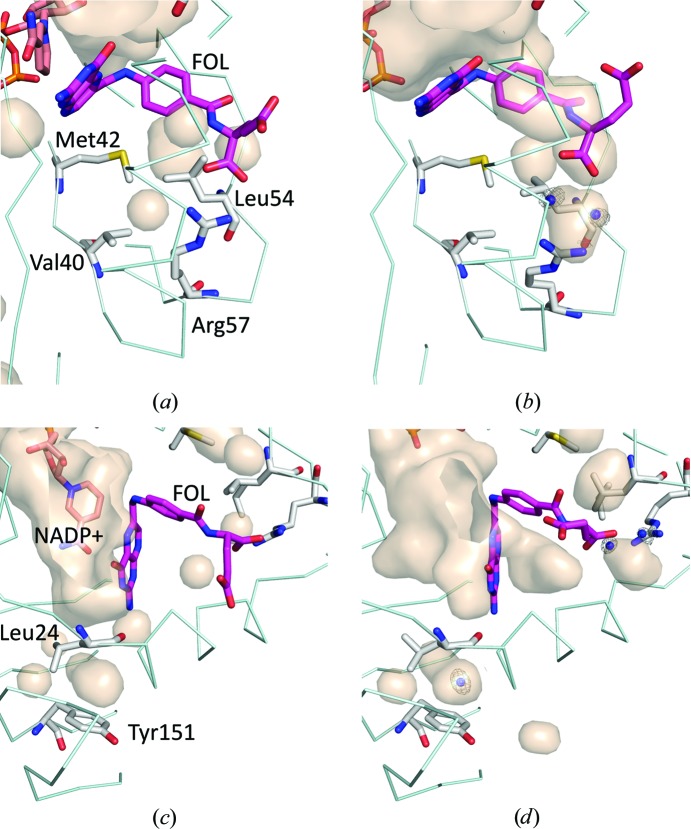
Magnified view of the internal cavities of the M20-open ecDHFR crystal. (*a*) 0.1 and (*b*) 750 MPa structures showing cavities near Leu54. The cavity surrounded by Val40, Met42 and Leu54 disappeared at 750 MPa. (*c*) 0.1 and (*d*) 750 MPa structures show a water molecule penetrating into a hydrophobic cavity above Tyr151. Cavities were drawn with the surface-cavity mode of *PyMOL* using a solvent radius of 1.2 Å. NADP^+^ and FOL are treated as components of the protein molecule, and water molecules are ignored. The electron density of the water is also drawn at 1.5σ. The 0.1 MPa structure is drawn using the *B* chain.

Some cavities shrank sufficiently to become undetectable using the 1.2 Å radius solvent probe as the pressure increased. For example, the volume of the cavity beside Val40, Met42 and Leu54 exhibited a precipitous decline between 270 and 500 MPa in the M20-open crystal. This shrinkage mainly resulted from a conformational change of the Leu54 side chain, as shown in Figs. 3 ▸(*a*) and 3 ▸(*b*). In fact, the distances between the C^α^ atoms of Met42 and Leu54, of Leu54 and Arg57, and of Val40 and Arg57, which surround the cavity, did not change significantly. The distances were 11.1 and 10.9 Å, 6.2 and 6.4 Å, and 6.7 and 7.0 Å at 0.1 and 750 MPa, respectively. On the other hand, the cavity above Tyr151 in the M20-open crystal did not shrink monotonically with pressure. Its volume apparently increased between 500 and 750 MPa by the penetration of a water molecule (Figs. 3 ▸ *c* and 3 ▸ *d*), and the side chain of Leu24 flipped coinciding with water penetration. The apparent increase in the total cavity volume and the molecular volume at higher pressure, as shown in Fig. 2 ▸, might be ascribed to water penetration into cavities.

### 3.2. Pressure-induced structural changes   

**Figure 4:**
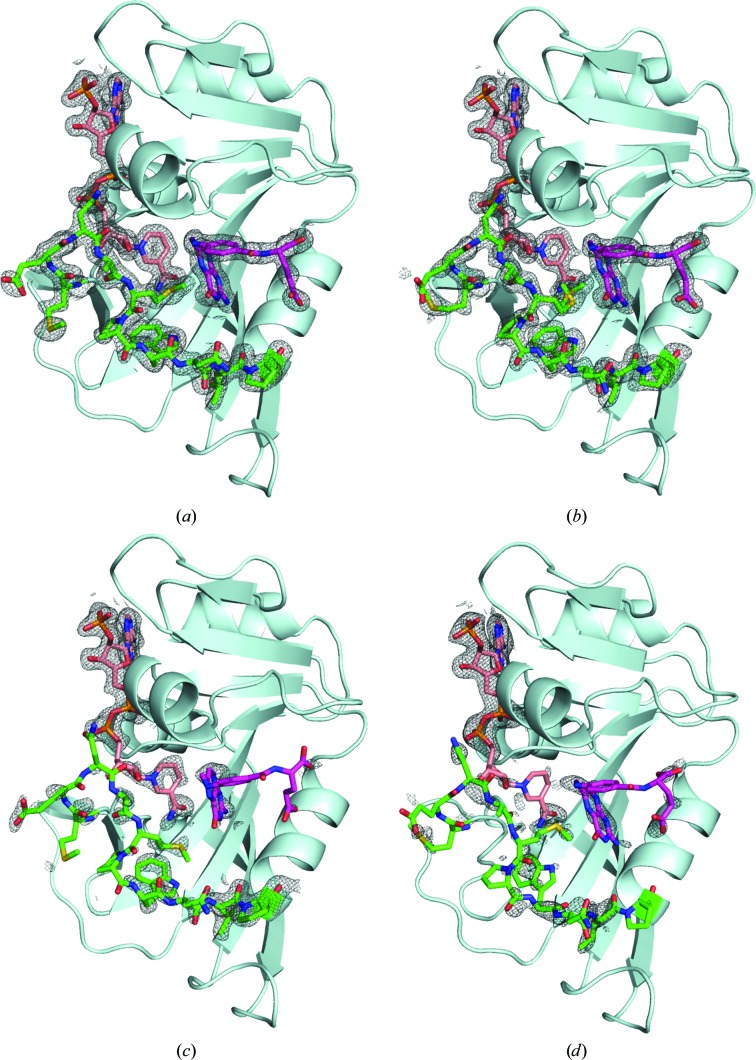
Electron-density maps near the M20 loop, NADP^+^ and FOL of the M20-closed crystal at (*a*) 0.1, (*b*) 400, (*c*) 650 and (*d*) 800 MPa. Electron density for the M20 loop and FOL are evident up to 400 MPa but become obviously weaker at 650 MPa. The electron-density map that covers key structures is shown at 1.0σ.

**Figure 5:**
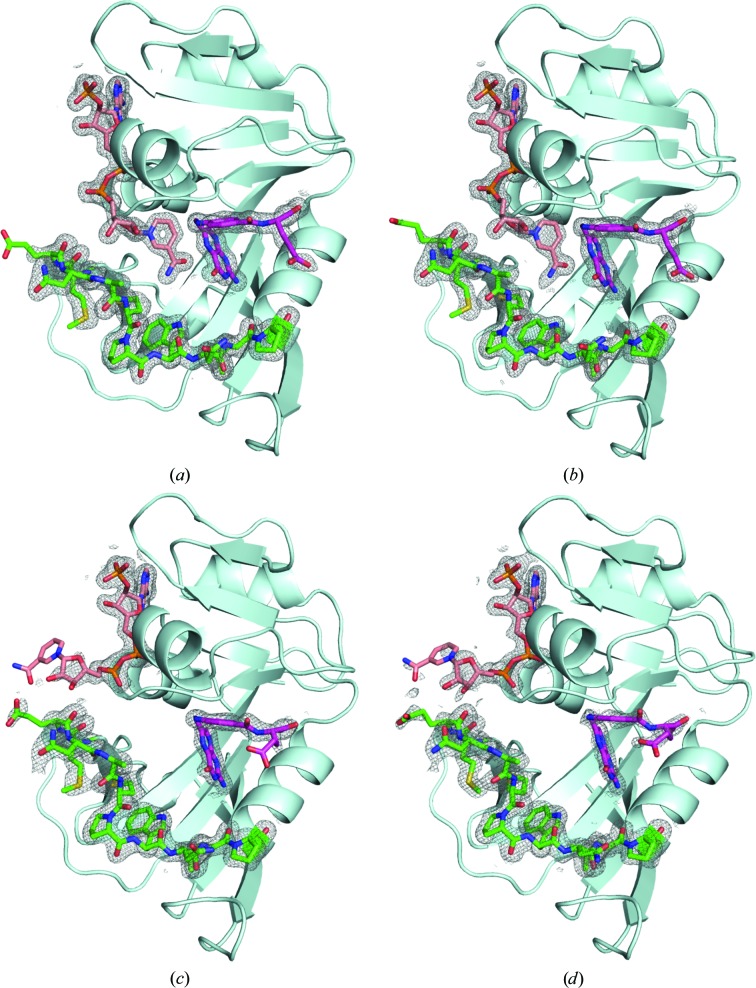
Electron-density maps near the M20 loop, NADP^+^ and FOL of the M20-open crystal at (*a*) 0.1, (*b*) 270, (*c*) 500 and (*d*) 750 MPa. The nicotinamide moiety of NADP^+^ is flipped out at 500 MPa, but the electron densities of the M20 loop and FOL are still evident at 750 MPa. The electron-density map covers key structures and is shown at 1.0σ. The structures in (*a*) and (*b*) are of the *B* chain.

**Figure 6:**
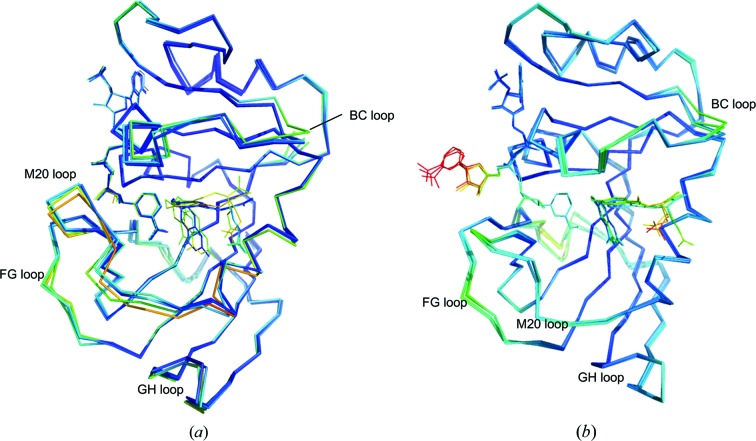
Overall structure changes of the (*a*) closed and (*b*) open conformation with pressure. All closed 0.1, 220, 400, 650 and 800 MPa, and open 0.1, 270, 500, 660 and 750 MPa structures are superimposed. The *B* chain was used to draw the structures of M20-open crystals at 0.1 and 270 MPa. Structures are colored as a rainbow based on the temperature factor, from a minimum of 5 Å^2^ to a maximum of 90 Å^2^.

The overall conformational changes of ecDHFR with pressure are presented in Figs. 4 ▸ and 5 ▸, which show the electron-density maps around the M20 loop, NADP^+^ and FOL. In the M20-closed crystal, the M20 loop was confirmed to be in the closed conformation from 0.1 to 400 MPa; NADP^+^ and FOL were also in their proper positions. At 650 MPa, a noteworthy decrease in the electron density was observed for the Met16–Asn23 portion of the M20 loop. Furthermore, when the pressure was increased to 800 MPa the region of the M20 loop that lost electron density extended to Trp30. The BC, FG and GH loops also became flexible as the pressure increased (Fig. 6 ▸ *a*). In addition to the loop region, the electron densities for FOL and the nicotinamide moiety of NADP^+^ decreased as the pressure increased (Figs. 4 ▸ *c* and 4 ▸ *d*). The average *B* factors of FOL were 47 and 56 Å^2^ at 650 and 800 MPa, respectively. When we tried to refine the occupancy of FOL, it became 70% or less with high *B* factors. On the other hand, the M20 loop of the M20-open crystal was more stable. Even at the highest pressure of 750 MPa, electron density in the M20 loop still clearly covered the structure (Fig. 5 ▸ *d*). Interestingly, at pressures above 500 MPa the nicotinamide moiety of NADP^+^ flipped and projected into the solvent region through a rotation about the PN—O3 bond. FOL also fluctuated above 500 MPa, but was more stable than in the M20-closed crystal. The average *B* factor for FOL was still as low as 45 Å^2^ at 750 MPa, and its occupancy was 76% when we refined it. Movements of the BC and FG loops were also observed at high pressure; this contrasted with the relatively stable M20 and GH loops (Fig. 6 ▸ *b*). Displacement of the FG loop following the flip of the NADP^+^ nicotinamide moiety appeared to represent the dominant contribution to the phase transition.

**Figure 7:**
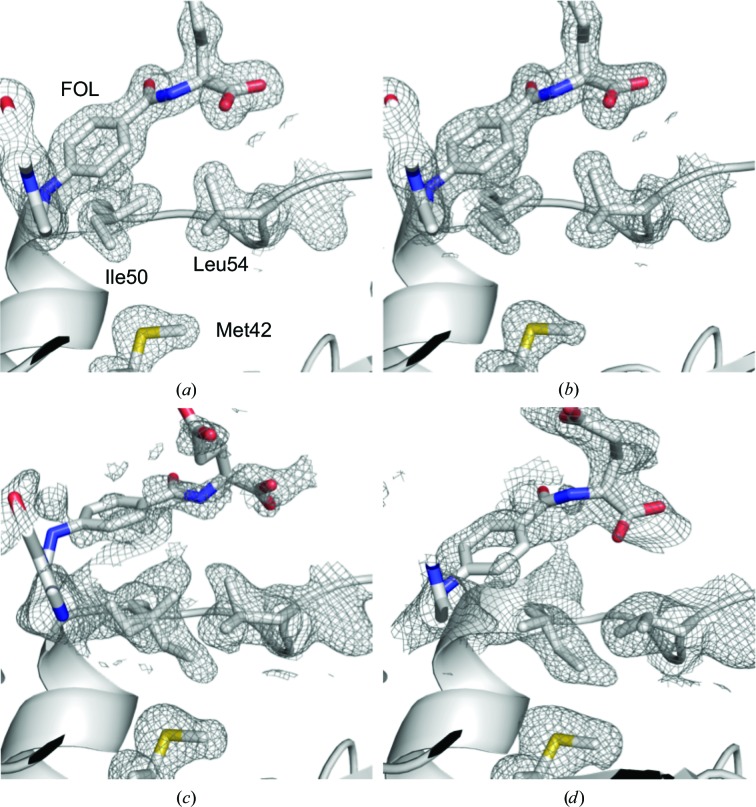
Structure change of Ile50 and Leu54 in the M20-closed crystal at (*a*) 0.1, (*b*) 400, (*c*) 650 and (*d*) 800 MPa. In (*c*), the side chain of Ile50 is modeled with two conformations. The electron-density maps cover key structures and are shown at 1.0σ in (*a*) and (*b*) and 0.5σ in (*c*) and (*d*).

At the FOL binding site of the M20-closed crystal, a conformational change of the Ile50 side chain was observed (Fig. 7 ▸). The side chain fluctuated at 650 MPa and completely flipped at 800 MPa. Side-chain flipping of Leu54 was also observed in the M20-open crystal, but the change for Ile50 was only observed in the M20-closed crystal.

### 3.3. Hydration structure   

**Figure 8:**
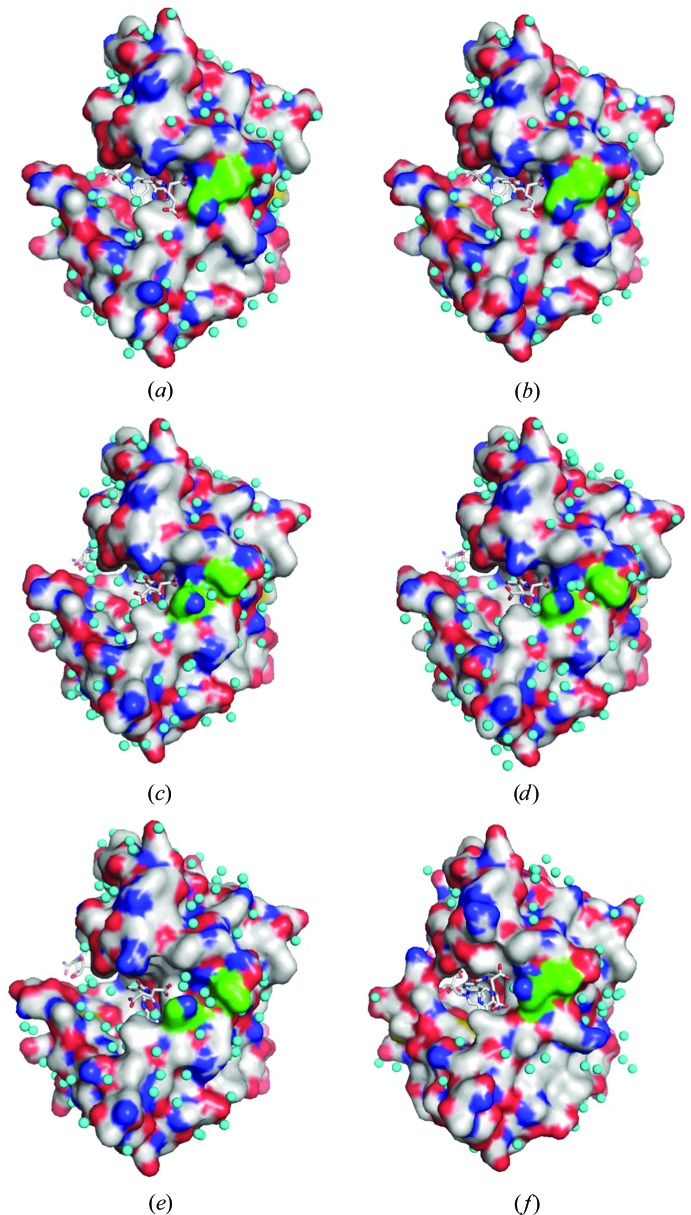
Surface-hydration water molecules of the M20-open crystal at (*a*) 0.1, (*b*) 270, (*c*) 500, (*d*) 660 and (*e*) 750 MPa. The 0.1 and 270 MPa structures are of the *B* chain. The same surface of the M20-closed crystal at 650 MPa is also shown in (*f*). Water molecules are shown as cyan spheres. FOL and NADP^+^ are shown as stick models. The surface at Lys32 and Leu36 is colored green.

Generally, the number of observed hydration water molecules increases with pressure (Nagae *et al.*, 2012 ▸; Yamada *et al.*, 2015 ▸); this tendency was further confirmed in this study. For the M20-closed crystal, the numbers of water molecules assigned were 87, 135, 134, 108 and 41 at 0.1, 220, 400, 650 and 800 MPa, respectively. The M20-open crystals contained (110, 107), (85, 88), 130, 137 and 134 water molecules at 0.1, 270, 500, 660 and 750 MPa, respectively (the pairs of values in parentheses indicate the numbers for the *A* and *B* chains in the asymmetric unit). The M20-closed crystal at 800 MPa contained a lower number of water molecules because the resolution decreased to as low as 2.2 Å owing to large movement of the loops and cofactors. The low number of water molecules in the M20-open crystal at 270 MPa may be related to the phase transition. The distribution of the surface-hydration water molecules in the M20-open crystal is shown in Fig. 8 ▸. It is noteworthy that the water molecules began to penetrate between Lys32 and Leu36 at 500 MPa, as highlighted by the green-colored surface in the figure. However, the same phenomenon was not observed in the M20-closed crystal (Fig. 8 ▸ *f*).

**Figure 9:**
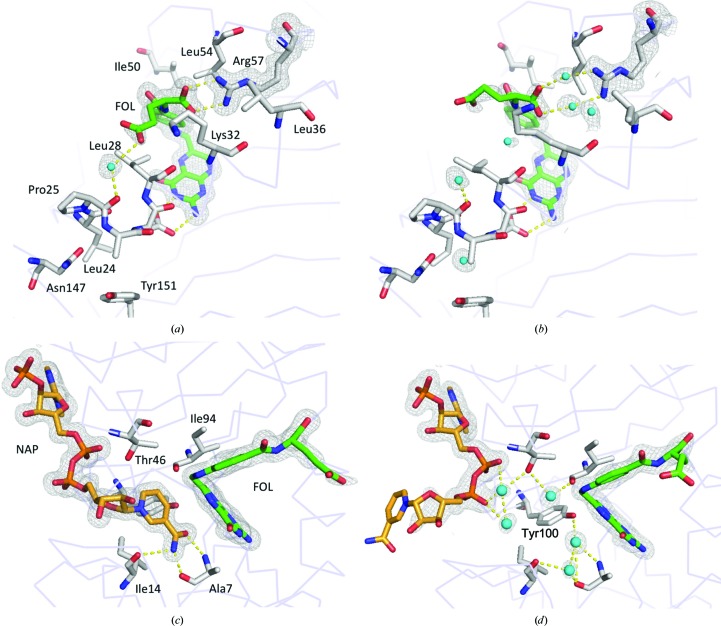
Water penetration and conformation changes near FOL and NADP^+^ in the M20-open crystal. (*a*) 0.1 and (*b*) 750 MPa structures at FOL and Arg57 and (*c*) 0.1 and (*d*) 500 MPa structures at FOL and NADP^+^. The 0.1 MPa structures are of the *B* chain. The electron-density map covers key structures and water molecules and is shown at 1.0σ.

In the M20-open crystal, interesting water behavior at the active site was observed as the pressure increased, as shown in Fig. 9 ▸. The guanidinium moiety of Arg57 directly interacted with the carboxyl group of FOL between 0.1 and 660 MPa. In contrast, this direct ligand–enzyme interaction changed to a water-mediated interaction at 750 MPa. The distance between the N^η1^ or N^η2^ atoms of Arg57 and the O1 or O2 atoms of FOL increased to 4.7–5.2 Å at 750 MPa with a conformational change of the BC loop (residues 51–57; Fig. 6 ▸ *b*), and two water molecules were clearly observed between Arg57 and FOL. These water molecules were located in the hydrogen-bonding network of the water molecules penetrating between Lys32 and Leu36 at high pressure (Fig. 8 ▸).

As shown in Fig. 5 ▸, a conformational change of NADP^+^ was also observed after the phase transition of the M20-open crystal. Below 270 MPa, the nicotinamide moiety of NADP^+^ made van der Waals contacts with the pteridine moiety of FOL. Above 500 MPa, the nicotinamide ribose moiety exited the binding site through a rotation of the PN—O3 bond. The water molecules that previously interacted with the pteridine moiety of FOL replaced FOL in the binding site (Figs. 9 ▸ *c* and 9 ▸ *d*). The water molecules hydrogen-bonded to Thr46 and Tyr100 resembled the waters observed in the ddTHF–NADPH product-analog complex (PDB entry 1rx6). Nevertheless, the M20 loop of the latter was in the occluded conformation (Sawaya & Kraut, 1997 ▸).

## 4. Discussion   

### 4.1. Pressure responses of the internal cavities   

As shown in Fig. 2 ▸, the molecular volumes were reduced by pressure according to Le Chatelier’s principle. By using HPPX, direct observation of the behavior of internal cavities of protein molecules is possible (Figs. 1 ▸ and 3 ▸). For partial molar volume reduction, accepting a water molecule into internal cavities is also effective (Collins *et al.*, 2005 ▸). In the case of ecDHFR, a water molecule penetrated into the cavity above Tyr151 at 750 MPa (Fig. 3 ▸ *d*). Such a phenomenon was also observed in our previous HPPX studies on IPMDH and hen egg-white lysozyme (Nagae *et al.*, 2012 ▸; Yamada *et al.*, 2015 ▸). The water molecule seems to interact with Tyr151 *via* a lone pair–π interaction, as previously observed in our lysozyme study, where Trp was the aromatic residue (Yamada *et al.*, 2015 ▸). Interestingly, this water penetration into the cavity was not observed in the M20-closed crystal even at 800 MPa.

Volume reduction of internal cavities is sometimes related to side-chain conformational changes in the surrounding amino acids. At the cavity beside the FOL binding pocket, the Leu54 side chain flipped at 750 MPa in the M20-open crystal (Fig. 3 ▸ *b*). Leu54 is an important residue for the hydride-transfer reaction of ecDHFR. Its van der Waals interaction with the FOL in the Michaelis complex dictates the orientation of the *p*-aminobenzoate moiety (Liu *et al.*, 2013 ▸). This structural change of Leu54 seems to relate to the substrate-recognition process of Arg57, which is on the same loop between αC and βC (Figs. 9 ▸ *a* and 9 ▸ *b*). The other residue at the cavity, Met42, is also important for ecDHFR activity. Met42 is a known dynamic communication hub of ecDHFR and functions as an allo­steric modulator (Mauldin & Lee, 2010 ▸). Mutation of Met42 to a bulky side chain may reduce the volume of the cavity and might have an influence on the mobility of the side chains of the residues around it, such as Leu54, and also on the reaction dynamics of ecDHFR. An M42W mutation reduced the flexibility of the GH loop, which is not in direct contact with the residue (Roston *et al.*, 2014 ▸). M42W/G121V mutations also affected the M20 loop dynamics (Fan *et al.*, 2013 ▸). The *K*_m_ and *k*_cat_ kinetic parameters of ecDHFR significantly increased in the M42W mutant (Ohmae *et al.*, 2005 ▸).

### 4.2. Pressure effect on the loop dynamics   

The M20 loop of ecDHFR adopts three conformations (open, closed and occluded) and is important for its enzymatic activity (Fierke *et al.*, 1987 ▸; Sawaya & Kraut, 1997 ▸). Recently, it has been reported that the M20-closed loop conformation affects the hydration of the active site and is related to the p*K*_a_ of the N5 atom of the substrate (Mhashal *et al.*, 2017 ▸).

In a high-pressure NMR study of ecDHFR, the structure of the M20 loop was noted to be pressure-sensitive (Kitahara *et al.*, 2000 ▸). The maximum pressure in the NMR experiment was 200 MPa, but it is known that the pressure response of protein molecules in crystals shifts to high-pressure regions (Katrusiak & Dauter, 1996 ▸; Hamajima *et al.*, 2016 ▸). Therefore, in our HPPX experiments we pressurized the M20-open and M20-closed crystals up to 750 and 800 MPa, respectively. Thus, we succeeded in observing M20 loop dynamics even in the crystal state.

In the M20-closed crystal, the loop moved with increasing pressure. The electron density of the loop became faint at and above 650 MPa. In contrast, the loop remained rather stable in its original position at high pressure in the M20-open crystal (Figs. 4 ▸ and 5 ▸). This pressure response of the M20 loop is obviously different to that of the main body of ecDHFR. Conformational motions of the loop are induced much earlier than other parts of the structure. Recently, computational studies on the M20 loop that were performed with pressure as a parameter showed that higher pressures would favor the open conformation (Huang *et al.*, 2017 ▸). Our HPPX results directly confirmed that the M20 loop favored the open conformation in its high-energy substate.

Pressure-induced motions of other loops were also observed (Fig. 6 ▸). The average *B* factor of the entire protein molecule was reduced by pressure, but the *B* factor increased in some of the loop regions. For example, movement at residues between 128 and 133 in the FG loop can be seen in both the M20-open and M20-closed crystals. In the M20-closed crystal at 800 MPa, conformational motions near residue 121 were also observed. Mutation of Gly121 to Val significantly reduces the activity of ecDHFR (Cameron & Benkovic, 1997 ▸), and a coupled motion network of Gly121, Met42 and Phe125 has been proposed (Singh *et al.*, 2015 ▸). A conformational change in the side chain of Val119 between the Michaelis model complex and the product ternary complex has also been discussed (Tuttle *et al.*, 2013 ▸). The pressure-induced conformational motions of the loop regions observed in our HPPX results may be related to these phenomena.

### 4.3. Structural change of NADP^+^ and folate   

We succeeded in directly observing the NADP^+^ nicotin­amide moiety flip in the crystal state using the M20-open crystal (Figs. 5 ▸, 6 ▸ and 9 ▸). This conformation of NADP^+^ was identical to the high-energy conformation minor state detected in an NMR relaxation dispersion experiment (Boehr *et al.*, 2006 ▸). We also observed a halfway structure for FOL recognition of Arg57 in the M20-open crystal. As shown in Fig. 9 ▸(*b*), two water molecules are located between FOL and Arg57. The structural change of Arg57 and these entering water molecules has not been observed, even in ensemble models calculated using structures collected at room temperature (Keedy *et al.*, 2014 ▸). The structural change induced by increasing pressure can be thought of as representing the ecDHFR Michaelis complex-formation process in the reverse order.

The fluctuation of FOL pertains to the ecDHFR product-release process. In the M20-closed crystal FOL was stable until 400 MPa, but its electron density became faint at 650 MPa (Fig. 4 ▸) and its occupancy could be refined to about 0.7 using *phenix.refine*. In the M20-closed crystal the nicotinamide moiety of NADP^+^ remained in the active site even at 650 MPa. The structural change of NADP^+^ was studied not only with respect to the hydride-transfer mechanism, but also the product-release process. Product release from ecDHFR is known to be cofactor-mediated, and the position of the nicotinamide moiety of NADP^+^ is important (Oyen *et al.*, 2015 ▸, 2017 ▸). In our HPPX results, the degree of FOL fluctuation was higher in the M20-closed crystal than in the M20-open crystal, in which the nicotinamide moiety was no longer adjacent to FOL at high pressure (Fig. 5 ▸). These results also show that ecDHFR product release, mimicked by FOL in our case, was faster when NADP^+^ did not flip the nicotinamide moiety out of the active site.

In the M20-closed crystal, conformational changes for Ile50 and Leu54 were observed above 650 MPa and coincided with the increased fluctuation of FOL (Fig. 7 ▸). These conformation changes of Ile50 and Leu54 accompanying FOL fluctuation seem to represent a direct observation of the ‘closed excited state’, which has previously been proposed based on ^1^H NMR chemical shift differences (Oyen *et al.*, 2017 ▸). When the side chain of Ile50 flips, the interaction between Ile50 and Met42 becomes larger and the hydrophobic interaction between Ile50 and the *p*-aminobenzoylglutamate moiety of FOL becomes weaker, inducing release of FOL from the pocket. On the other hand, in the M20-open crystal, where the FOL fluctuation was lower than in the M20-closed crystal, only Leu54 changed its conformation (Fig. 3 ▸ *b*) and structural change of Ile50 was not observed.

## 5. Conclusions   

High-energy substates related to a reaction cycle cannot be detected using traditional crystallographic methods because of their low frequency in the population at atmospheric pressures (Collins *et al.*, 2011 ▸; Wan, Bennett *et al.*, 2014 ▸). Many methods exist to study these states, but we believe that X-ray crystallography is the best method because it can directly observe three-dimensional molecular structures, including water molecules. Traditional crystallography is not suited to studying dynamics because it uses a crystal, which can only yield information on the averaged low-energy structures of the molecules within it. We have demonstrated the possibility of using HPPX as a method of capturing the high-energy substates, or the transient structures, related to the reaction cycle of protein molecules. For ecDHFR, our data were consistent with previous results, such as NMR relaxation dispersion experiments and computational simulations. The development of more effective HPPX experiment systems, for example systems that collect diffraction data easily at fine pressure intervals, may enable the production of three-dimensional movies of high-energy protein substates.

## Supplementary Material

PDB reference: ecDHFR, M20-open, 0.1 MPa, 4x5f

PDB reference: 270 MPa, 4x5g

PDB reference: 500 MPa, 4x5h

PDB reference: 660 MPa, 4x5i

PDB reference: 750 MPa, 4x5j

PDB reference: M20-closed, 0.1 MPa, 5z6f

PDB reference: 220 MPa, 5z6j

PDB reference: 400 MPa, 5z6k

PDB reference: 650 MPa, 5z6l

PDB reference: 800 MPa, 5z6m

## Figures and Tables

**Table:** (*a*) M20-open crystal. *HKL*-2000 and *REFMAC* were used to process diffraction images and perform structure refinements, respectively.

PDB code	4x5f	4x5g	4x5h	4x5i	4x5j
Data collection					
Pressure (MPa)	0.1	270	500	660	750
Diffraction source	FR-E SuperBright	NW12A, PF-AR	NW12A, PF-AR	NW12A, PF-AR	NW12A, PF-AR
Wavelength (Å)	1.54	0.75	0.75	0.71	0.71
Space group	*P*2_1_	*P*2_1_	*C*2	*C*2	*C*2
Unit-cell parameters (Å, °)	*a* = 38.96, *b* = 59.93, *c* = 72.32, β = 102.81	*a* = 38.72, *b* = 59.37, *c* = 71.70, β = 102.54	*a* = 74.18, *b* = 58.64, *c* = 38.28, β = 106.78	*a* = 73.49, *b* = 58.52, *c* = 38.11, β = 107.01	*a* = 73.88, *b* = 58.59, *c* = 38.20, β = 107.10
Resolution (Å)	23.31–1.70 (1.73–1.70)	37.83–1.90 (1.93–1.90)	36.68–1.90 (1.93–1.90)	44.97–1.80 (1.83–1.80)	36.54–1.85 (1.88–1.85)
Observed reflections	141078	74935	52919	32396	67111
No. of unique reflections	35866	25274	12674	14675	13475
*R*_merge_ (%)	7.8 (34.0)	5.4 (14.7)	7.6 (43.0)	6.3 (49.7)	8.4 (48.3)
〈*I*/σ(*I*)〉	20.7 (2.4)	29.3 (9.1)	27.0 (4.8)	16.8 (1.9)	16.7 (2.3)
Completeness (%)	95.9 (80.1)	91.8 (95.0)	98.5 (99.8)	95.1 (94.8)	98.3 (100.0)
Multiplicity	4.1 (3.5)	3.2 (3.0)	4.2 (4.4)	2.3 (2.2)	5.1 (5.1)
Average mosaicity (°)	0.40	0.16	0.49	0.77	0.58
Refinement					
*R*_work_ (%)	15.23	15.47	16.02	17.07	20.30
*R*_free_ (%)	18.97	20.85	22.22	24.05	26.45
R.m.s.d., bonds (Å)	0.022	0.019	0.019	0.019	0.018
R.m.s.d., angles (°)	2.283	2.085	2.007	2.082	2.044
Molecules in asymmetric unit	2	2	1	1	1
No. of waters per molecule	110, 107†	85, 88†	130	137	134
Cruickshank DPI (Å)	0.10	0.16	0.16	0.15	0.18

**Table:** (*b*) M20-closed crystals. *XDS* and *PHENIX* were used to process diffraction images and perform structure refinements, respectively.

PDB code	5z6f	5z6j	5z6k	5z6l	5z6m
Data collection					
Pressure (MPa)	0.1	220	400	650	800
Diffraction source	BL2S1, AichiSR	BL2S1, AichiSR	BL2S1, AichiSR	BL2S1, AichiSR	BL2S1, AichiSR
Wavelength (Å)	0.75	0.75	0.75	0.75	0.75
Space group	*P*2_1_2_1_2_1_	*P*2_1_2_1_2_1_	*P*2_1_2_1_2_1_	*P*2_1_2_1_2_1_	*P*2_1_2_1_2_1_
Unit-cell parameters (Å)	*a* = 34.11, *b* = 45.91, *c* = 99.08	*a* = 34.13, *b* = 44.93, *c* = 98.44	*a* = 34.06, *b* = 44.53, *c* = 97.98	*a* = 34.13, *b* = 41.88, *c* = 97.93	*a* = 33.92, *b* = 41.85, *c* = 97.53
Resolution (Å)	41.66–1.80 (1.84–1.80)	49.22–1.80 (1.84–1.80)	48.99–1.80 (1.84–1.80)	48.96–1.90 (1.94–1.90)	48.76–2.20 (2.27–2.20)
Observed reflections	118259	134123	74631	102468	46374
No. of unique reflections	15050	14502	14269	11647	7493
*R*_merge_ (%)	10.9 (71.7)	7.3 (39.8)	8.2 (50.8)	10.5 (52.6)	10.9 (66.1)
〈*I*/σ(*I*)〉	11.6 (2.7)	22.3 (5.0)	14.5 (2.9)	14.0 (3.1)	15.9 (2.5)
Completeness (%)	100.0 (99.5)	99.4 (98.1)	99.0 (92.6)	99.8 (97.7)	99.6 (97.4)
Multiplicity	7.9 (7.9)	9.2 (8.2)	5.2 (4.8)	8.8 (6.5)	3.2 (2.6)
Average mosaicity (°)	0.18	0.08	0.11	0.23	0.34
Refinement					
*R*_work_ (%)	17.74	16.69	18.14	19.32	23.18
*R*_free_ (%)	21.01	20.30	21.43	23.28	30.59
R.m.s.d., bonds (Å)	0.007	0.007	0.007	0.007	0.009
R.m.s.d., angles (°)	1.175	1.143	1.150	1.085	1.156
Molecules per asymmetric unit	1	1	1	1	1
No. of waters per molecule	87	135	134	108	41
Cruickshank DPI (Å)	0.13	0.13	0.14	0.19	0.45

† Numbers assigned for the two DHFR molecules in the asymmetric unit.
